# Effects of Long-Term Non-Pruning on Main Quality Constituents in ‘Dancong’ Tea (*Camellia sinensis*) Leaves Based on Proteomics and Metabolomics Analysis

**DOI:** 10.3390/foods10112649

**Published:** 2021-11-01

**Authors:** Yiyong Chen, Bo Zhou, Jianlong Li, Hao Tang, Lanting Zeng, Qin Chen, Yingying Cui, Jiayu Liu, Jinchi Tang

**Affiliations:** 1 Tea Research Institute, Guangdong Academy of Agricultural Sciences & Guangdong Provincial Key Laboratory of Tea Plant Resources Innovation and Utilization, Dafeng Road 6, Tianhe District, Guangzhou 510640, China; chenyiyong@gdaas.cn (Y.C.); zhoubo@gdaas.cn (B.Z.); skylong.41@163.com (J.L.); tanghao@gdaas.cn (H.T.); cuiyingying@gdaas.cn (Y.C.); liujiayu@gdaas.cn (J.L.); 2 Key Laboratory of South China Agricultural Plant Molecular Analysis and Genetic Improvement & Guangdong Provincial Key Laboratory of Applied Botany, South China Botanical Garden, Chinese Academy of Sciences, Xingke Road 723, Tianhe District, Guangzhou 510650, China; zenglanting@scbg.ac.cn; 3 Chaozhou Tea Science Research Center, Chaozhou 512000, China; gdczchen@126.com

**Keywords:** *Camellia sinensis*, pruning, ‘Dancong’ tea, quality constitutes, catechins

## Abstract

‘Dancong’ tea is a famous traditional Oolong tea. In order to keep the original taste of “ancient tea trees”, most of the ‘Dancong’ tea plants are planted in a single plant pattern without pruning. The objective of this study was to explore the effects of long-term non-pruning on main quality constituents in ‘Dancong’ tea. The results showed that the contents of free amino acids, chlorophylls, and floral-honey aromatic substances in tea leaves of unpruned tea plants were higher than those in every year pruned tea plants, while the catechin content in leaves of pruned tea plants was higher than that in leaves of unpruned tea plants. Quantitative proteomics analysis showed that most enzymes involved in biosynthesis of catechins were downregulated in leaves of unpruned tea plants. Five proteins involved in chlorophyll metabolism and 12 proteins related to photosynthesis were upregulated, and the results suggested that higher chlorophyll content and more efficient photosynthetic energy conversion may be important for the higher accumulation of special quality components in leaves of unpruned tea plants. The findings of this study will advance our understanding of the mechanism of formation of different metabolites in leaves of unpruned and pruned tea plants.

## 1. Introduction

Tea leaves, in principle a bud with two adjacent leaves, are processed into different types of tea products. Due to its pleasant flavor and important health benefits, tea has become the second most consumed non-alcoholic beverage after water [1,2]. The unique taste and aroma of tea are closely related to the special compounds it contains; specifically, tea polyphenols, amino acids, aromatic compounds, pigments, and organic acids are important quality ingredients for tea leaves [3,4]. Catechins account for more than 70% of all polyphenols present in tea leaves. Catechins reportedly contribute to the astringency and bitterness of green tea infusions, while in oolong tea infusions, a sweet aftertaste follows after bitterness and astringency [5,6]. It has been reported that ester catechin content in tea leaves is much higher than non-ester catechins, while epigallocatechin gallate (EGCG) is the ester catechin present in the highest concentration in green tea [7,8,9]. Caffeine is another bitter tasting component in tea infusions, and the biosynthesis pathway of caffeine is part of purine metabolism [10,11]. Caffeine synthase is the crucial enzyme in the pathway, catalyzing the conversion of theobromine to caffeine [12]. Amino acids comprise another tea-quality determination component that contribute to the umami taste of tea. Amino acids found in tea are mainly a product of biosynthesis or of the degradation of proteins [13,14]. Chlorophylls, generally composed of chlorophyll a and chlorophyll b, are health beneficial components in tea leaves and are vital pigments involved in photosynthesis. Previously, Wei et al. (2011) reported that the concentration of chlorophylls is closely associated with catechin biosynthesis; specifically, chlorophyll a and chlorophyll b concentrations significantly affect (+)-catechin content [15]. Aromatic compounds contribute to flavor of tea; according to their metabolic origin, four kinds of aroma compounds (each arising from corresponding molecular pathways) are derived from tea: terpenoids, phenylpropanoids/benzenoids, carotenoids, and fatty acids [16]. Finally, different metabolite compositions may be formed under the influence of various factors, including, soil conditions, climate change, and cultivation practices.

Tea plants originated in China, and artificial cultivation of tea has a history of more than 3000 years. Efficient tea-cultivation technology to grow high yield and quality tea leaves has been explored by farmers throughout history. Management practices adopted by farmers are most important for plant growth and formation of high-quality constituents. Under different agronomic practices, such as the application of organic fertilizers, shade treatment, pruning or non-pruning of tea trees, different metabolite compositions will be formed based on metabolite contents [17,18]. Pruning is an essential, and the most significant, agronomic management practice, aimed to enhance branching and increase the number of tender leaves. Furthermore, compared with unpruned tea plants, it is more convenient for the farmer to pluck tea leaves in pruned tea plants every year. Several studies have reported the effects of pruning on tea quality and constituents. For example, the synthesis of indole-3-acetonitrile and menaquinone increased in pruned tea plants and enhanced shoot growth and development [17]. Similarly, EGCG concentration was much higher in pruned than in unpruned tea trees, and serine carboxypeptidase was upregulated for biosynthesis of EGCG in pruned tea plants [19]. Different concentrations of EGCG in long-term pruned and unpruned tea plants account for differences in the degree of bitterness and astringency in the corresponding tea products. Rubel Mozumder et al. (2020) reported that the different metabolic characteristics in tea leaves from pruned and unpruned plants were related to climatic conditions, and photosynthesis active in unpruned tea may increase the accumulations of catechins [20].

‘Dancong’ tea is a famous traditional Oolong tea, originating in Chaozhou city, Guangdong Province, has a long history of more than 700 years dated back to the Song Dynasty in China [21]. To keep the original taste of “ancient tea trees” and protect the healthy growth of tea trees, the growers never pruned the tea plant and allowed it to grow naturally. Pruning, as an essential and commonly cultivation management, is wildly performed in tea plantation every year to enhance leaf productivity. In recent years, some tea plantation began to trim the tea plants every year to improve the production efficiency of ‘Dancong’ tea. However, the original quality characteristics were lost. Long term non-pruning is considered to be closely related to the formation of the special quality constituents of ‘Dancong’ tea leaves. In the present study, we compared tea products from long-term unpruned and pruned tea plants, using sensory evaluation. Our results showed that the aroma and taste of the leaves from unpruned tea plants were superior to those of the leaves from pruned tea plants. Proteomics and metabolomics were used to explore the mechanism of formation of the different tea leaf-quality constituents in unpruned and pruned ‘Dancong’ tea trees.

## 2. Materials and Methods

### 2.1. Plant Materials and Sample Preparation

**Figure 1:**
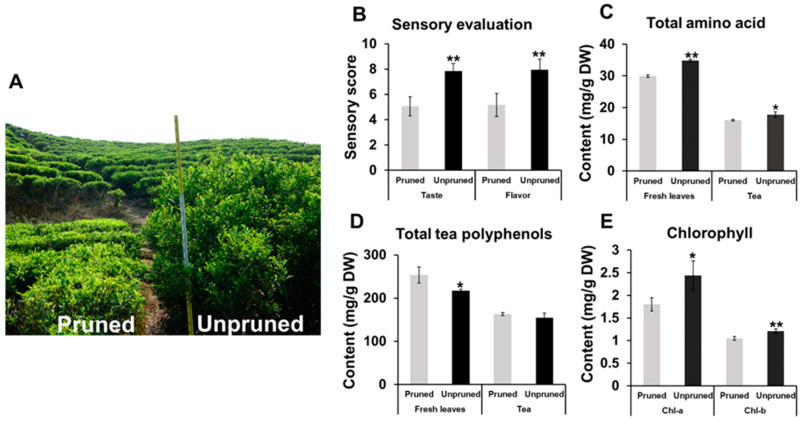
Phenotypes (**A**), sensory evaluation score (**B**), total contents of free amino acids (**C**), total contents of tea polyphenol (**D**), contents of chlorophylls (**E**) in tea leaves from long-term unpruned and pruned tea plants. Data is presented as mean ± SD. * represents significant difference (*p* < 0.05), ** represents extremely significant difference (*p* < 0.01).

The tea leaf samples were collected from thirty-two years old ‘Lingtou Dancong’ tea plants cultured in a tea plantation in Lingtou village, Chaozhou city, Guangdong Province, China. Pruned tea plants were pruned once every year to maintain plant height within 80–90 cm. Long-term unpruned tea plants were cultured in parallel under identical conditions and have never been pruned since the time they were planted, whereby their average height reaches over 260 cm (Figure 1A). Samples (the apical bud and two adjacent leaves) were collected in April 2019; tea leaves used for oolong tea manufacturing were transported to the factory immediately. Oolong tea was manufactured according to the local traditional processing technology [22]. Processed oolong tea products were subjected to sensory evaluation and aroma composition analysis. Fresh tea leaves used for determination of physicochemical properties and proteomic analysis; For all samples, three biological replicates were conducted. Samples were immediately frozen in liquid nitrogen after plucking and stored at −80 °C until use.

### 2.2. Sensory Evaluation (Organoleptic Experiments)

Sensory evaluation was performed at the Tea Research Institute, Guangdong Academy of Agricultural Sciences. Tea samples from pruned and unpruned tea plants were prepared, and then blind-coded with random numbers. Taste and flavor of the tea samples were evaluated as the method described in the national standard “Tea Sensory Evaluation Method (GB/T23776-2018)”. Tea samples (3 g) were extracted with 150 mL freshly boiled water for 5 min in a tea evaluation cup; then, the resulting tea infusion was filtered into an evaluation bowl. Twenty trained and experienced panelists participated in the evaluation, and a 10-point hedonic scale was employed to evaluate each tea-infusion sample.

### 2.3. Chlorophyll Assays

Fresh tea-leaf samples (0.2 g) were ground to a powder under liquid nitrogen in a mortar, and 10 mL extraction solution (80% acetone containing a small amount of CaCO_3_) was added to the mortar. Grinding continued until the tea powder turned white, following which it was filtered. Filtrates were analyzed using a Shimadzu UV-1800 spectrophotometer (Shimadzu, Kyoto, Japan), by measuring absorbance at 663 and 645 nm for each sample. Concentrations of chlorophyll a and chlorophyll b were calculated using the equation described by Lichtenthaler & Wellburn (1983) [23].

### 2.4. Concentration of Tea Polyphenols and Composition of Catechins and Caffeine

Frozen tea leaves were ground with a tissuelyser (JXFSTPRP-24, Shanghai Jingxin Industrial Development Co., Ltd., Shanghai, China). Then, each powder sample (0.2 g) was extracted with 5 mL of preheated 70% methanol solution for 10 min in a water bath at 70 °C. After centrifugation (3500 rpm, 25 °C, 10 min), precipitates were extracted again, and supernatants were collected and combined. Concentration of tea polyphenols was determined using the Folin-Ciocalteu assay, as described by Lai et al. (2016) [24]. Sample absorbance was recorded at 765 nm using a Shimadzu UV-1800 spectrophotometer.

The composition of caffeine and catechins was determined by HPLC. After filtering through a 0.45 µM Millipore filter, tea extracts were submitted to an Agilent 1200 Series HPLC System (Agilent Technologies, Santa Clara, CA, USA) equipped with a ZORBAX Eclipse Plus C18 column (5 µM, 4.6 mm × 250 mm). The injection volume was 10 µL, and the column temperature was maintained at 35 °C. Mobile phase A consisted in 0.05% (*v*/*v*) formic acid in deionized water, and mobile phase B was acetonitrile. Catechin separation was achieved using the following gradient elution sequence: 0–5 min, 72.5% A, 27.5% B; 30 min, 20% A, 80% B; 35 min, 72.5% A, 27.5% B, and 72.5% A, 27.5% B (*v*/*v*) until 40 min for re-equilibration. The flow rate was set at 1.0 mL/min. An Agilent DAD ultraviolet detector (280 nm) was used to detect peak intensities. Catechin, caffeine, epicatechin, epicatechin gallate, epigallocatechin, epigallocatechin gallate, gallic acid, gallocatechin, gallocatechin gallate were identified by comparison with authentic standards, calibration curves were used to quantitative analyses these compounds.

### 2.5. Free Amino Acid Content Analysis

Total amino acid content in tea leaves was determined using the ninhydrin method described by Chen et al. (2017) [25]. Absorbance was recorded at 570 nm using a spectrophotometer.

Free amino acid contents were determined following the method described previously [26]. Finely powdered tea leaves (1.0 g) were extracted with 10 mL of 10% formic acid in methanol. After centrifugation (12,000 rpm, 10 min), the resulting supernatants were filtered and 5 μL of each sample was submitted to ultra-performance liquid chromatography–tandem mass spectrometry (UPLC-MS/MS). Waters (Milford, MA, USA) Acquity Ultra Performance LC equipped with an AB 4000 triple quadrupole mass spectrometer was used to detect free amino acids.

### 2.6. Analysis of Tea Volatiles

Aroma compounds in fresh tea leaves and tea products were analyzed as per the method described by Zeng et al. (2019) [27]. To extract the aroma compounds, 0.5 g tea-leaf sample powder was extracted using 2 mL dichloromethane in a 5 mL glass vial, with 0.5 nmol ethyl n-decanoate added as an internal standard. After removal of the residual water through a sodium sulphate anhydrous column, the extraction solution was collected. GC-MS QP2010 SE (Shimadzu Corporation, Kyoto, Japan) equipped with A SUPELCOWAX 10 column (30 m × 0.25 mm × 0.25 μm, Supelco Inc., Bellefonte, PA, USA) was used to analyzed aroma compounds present in the samples. Mass spectra collection was scanned within the range from *m*/*z* 40 to *m*/*z* 200. Identified and quantitated volatile compounds were direct compared with authentic standards and calibration curves. Methyl salicylate, phenylacetaldehyde, linalool oxide, benzyl alcohol,1-Hexanol, (E)-nerolidol, (Z)-3-hexenol, (Z)-3-hexenyl acetate, linalool, geraniol, α-farnesene, benzaldehyde were available authentic standards in the lab, and the other comparison without authentic standards were identified with retention indices (RI) [27].

### 2.7. Protein Extraction, Digestion, Tandem Mass Tag (TMT) Labeling, and Mass Spectrometry

Total proteins were extracted from fresh tea-leaf tissue of pruned and unpruned tea plants, as previously described (Chen et al., 2017) [25]. Finely powdered samples (3.0 g) were extracted overnight at −20 °C with extraction buffer (TCA/acetone 1:9, containing 65 mM DTT). Protein pellets were collected by centrifugation (6000 rpm, 20 min), then washed two times with cold acetone, each time for 2 h, and centrifuged at 12,000 rpm for 20 min. Protein pellets were dissolved in 150 mM Tris-HCl buffer (4% SDS, pH 8.0) and determined content. After that, protein samples (100 μg) were digested and desalted. STD buffer (4% SDS, 100 mM Tris-HCl pH 8.0, and 100 mM DDT solution) 30 μL was added to 200 μg of tea leaves protein sample, incubated in boiling water for 5 min to dissolve the protein. Addition of 200 μL of UA buffer (800 mM urea, 150 mM Tris-HCl, pH 8.0) after cooled to room temperature, and then subjected to a 10 kD filter (Sartorius, German), centrifuged at 14,000 g for 15 min. Washed filter with 200 μL of UA buffer again. Then, 100 μL UA buffer containing 100 mM iodoacetamide was added, and then incubated under darkness for 30 min. After centrifugation (14,000 g, 15 min), the filters were washed twice with 100 μL of UA buffer, and then washed with 100 μL of 50 mM triethylammonium bicarbonate (pH 8.5) twice. Finally, the samples were digested with 40 μL of trypsin (Promega, Madison, WI, USA) buffer (2 μg trypsin in 40 μL dissolution buffer) overnight at 37 °C. The resulting peptides were collected by centrifugation. After labeling with TMT reagents (Thermo Scientific, San Jose, CA, USA) according to the protocol, a high pH reversed-phase peptide fractionation kit was used to fractionate peptides. Fractions were collected for 8–60 min, and eluents were collected in centrifugal tubes 1–15 every min, in turn. Samples were recycled in this order until the end of the elution gradient. Separated peptides were lyophilized for mass spectrometry (MS) analysis. Peptide fractions were analyzed by nano LC-MS/MS followed by MS/MS in a Q EXACTIVE (Thermo Fisher Scientific, San Jose, CA, USA) equipped with nanoelectrospray ionization. Each graded sample was separated by HPLC liquid system Easy nLC at a nanoliter flow rate. The chromatographic column was balanced with 95% liquid A (0.1% formic acid aqueous solution), and the samples were loaded to the loading column by automatic sampler (Thermo Scientific Acclaim PepMap100, 100 μm × 2 cm, nanoViper C18), solution B was 0.1% formic acid acetonitrile aqueous solution (acetonitrile 84%). The separation was performed on a Thermo Scientific EASY Column (10 cm, ID75 μm, 3 μm, C18-A2) at a flow rate of 300 nL/min. Separated samples were analyzed by Q-EXactive mass spectrometer. The detection mode is positive ion, and the scanning range of parent ion is 300–1800 *m*/*z*, the resolution of primary mass spectrometry is 70,000 at 200 *m*/*z*, the Automatic Gain control (AGC) target is 1e6, and the Maximum IT is 50 ms. The dynamic exclusion time was 60 s. The mass charge ratios of peptides and peptide fragments were collected as follows: 20 fragment profiles (MS2 Scan) were collected after each full scan. MS2 Activation Type was HCD, and Isolation Window was 2 *m*/*z*. Normalized Collision Energy is 30 eV, and ms resolution is 17,500 at 200 *m*/*z*, Underfill is 0.1%.

### 2.8. Sequence Database Search and Data Analysis

Protein and peptide identification was performed using Mascot 2.2 software and Proteome Discoverer1.4 (Thermo Fisher Scientific, San Jose, CA, USA). The C. sinensis protein database (30,052 protein, https://www.uniprot.org/proteomes/UP000306102, accessed on 26 February 2021) was used for peptide mapping and protein identification. Search parameters were as described by Chu et al. (2015) [28]. Peptides with false discovery rate ≤1% were excluded. For protein differential analysis, increased protein expression or downregulated protein species were determined with a 1.2-fold cutoff and a *p* value < 0.05. All differentially expressed proteins were mapped to Gene Ontology (GO) database for functional annotation. Kyoto Encyclopedia of Genes and Genomes (KEGG) pathway (http://www.genome.jp/kegg/, accessed on 20 April 2021) enrichment analysis was conducted using a KEGG Automatic Annotation Server software.

### 2.9. Quantitative Real Time Polymerase Chain Reaction (qRT-PCR) Analysis

To further evaluate the reliability of the TMT-proteomics results, catechin biosynthesis-related genes were selected for qRT-PCR analysis. A Quick RNA isolation Kit (Huayueyang Biotechnology Co., LTD., Beijing, China) was used to isolated the total RNA from fresh tea leaves. First strand cDNAs were synthesized using the Reverse Transcription kit reagents (TaKaRa, Tokyo, Japan). The qRT-PCR reactions were performed using SYBR^®^ Green Pro Taq HS Kit (Accurate Biotechnology Co., Ltd., Changsha, China) in a 20-μL reaction system. Roche LightCycle 480 (Roche Applied Science, Mannheim, Germany) was used to perform the qRT-PCR reaction. The 2^−ΔΔCt^ method was used to calculate relative expression levels. The sequences of the primers used are listed in Table S1. *CsEF-1α* was used as an internal gene reference [29]. Changes in mRNA level of the test gene for each treatment were normalized to that of *CsEF-1α*.

### 2.10. Statistical Analysis

Statistical analysis was conducted using the SPSS statistical package (version 18.0) and Microsoft Excel 2016. Data of three independent replicates of each group are shown as the mean ± standard deviation. Student’s test was used to determine the differences among the various treatment groups. Statistical significance was defined as a *p* value less than 0.05.

## 3. Results and Discussion

### 3.1. Tea Leaves from Unpruned Tea Plants Were Superior in Taste and Flavor and Higher in Total Amino Acid and Chlorophyll Contents

Sensory characteristics of tea prepared with leaves from long-term unpruned and pruned tea plants was evaluated in terms of taste and flavor. The scores of tea infusions prepared with leaves from long-term unpruned tea plants were much higher than those obtained by infusions prepared with leaves of pruned plants; furthermore, all professional tea tasters consistently evaluated unpruned tea leaves as better in terms of aroma and taste (Figure 1B). Although there was no significant difference between the contents of total amino acids and polyphenols in tea products, total amino acid contents in fresh leaves from unpruned tea plants (34.84 ± 0.39 mg/g DW) were significantly higher than those in leaves from long-term pruned tea plants (29.96 ± 0.43 mg/g DW). Conversely, total polyphenol content was lower in leaves from unpruned plants than that of fresh leaves from long-term pruned tea plants (Figure 1C,D). The difference in taste and flavor between tea products may be related to the transformation of different contents of prerequisite substances, such as amino acids and polyphenols, during tea processing [13,30]. Chlorophyll a and chlorophyll b contents in fresh leaves from long-term unpruned tea plants were higher than those from pruned plants (Figure 1E). Compared with that pruned annually, non-pruning tea plants have phenotypes that higher height and greater canopy area to receive light. Increased chlorophyll content can promote photosynthesis in leaves, which, in turn, will promote sink-source alterations. It has been reported that chlorophyll content was significantly correlated with individual catechin content, but not with total catechin, and chlorophyll a played the key role [15]. The results in this study may indicate that higher tree height and more branching structure in unpruned tea plants are more conducive to the synthesis of tea quality components in leaves.

### 3.2. Analysis of Differentially Expressed Proteins between Leaves from Long-Term Unpruned and Pruned Tea Plants

**Figure 2:**
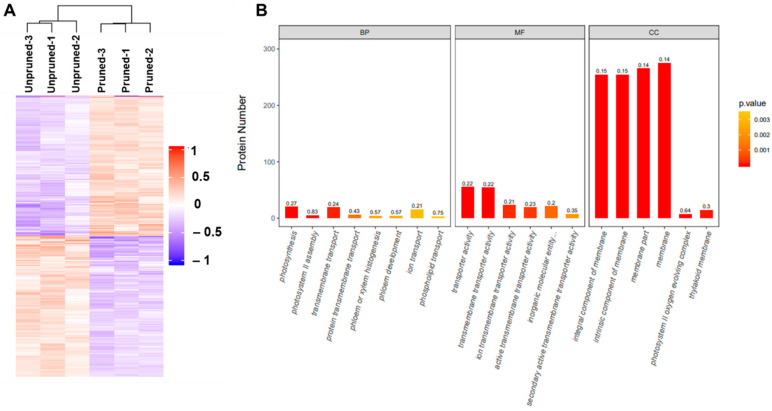
Hierarchical cluster (**A**) and gene ontology classification (**B**) for differentially expressed proteins in leaves of pruned versus unpruned tea plants. The ordinate indicates the number of differential proteins for each functional classification, the color of the bar chart represents the significance of enriched GO functional classification, the color gradient represents the size of *p* value. The label at the top of the bar chart shows the enrichment factor (richFator ≤ 1), which represents the proportion of the number of differentially expressed proteins annotated to a GO functional class to the number of all proteins identified to that GO functional class. BP, biological process; MF, molecular function; CC, Cellular Component.

In this study, 132,187 spectra, 54,032 peptides, and 9135 proteins were identified in ‘Lingtou Dancong’ tea leaves. For the analysis of the differentially expressed proteins (DEPs), proteins showing greater than 1.2-fold change and *p* < 0.05 were considered as DEPs. In leaves of long-term unpruned tea plants, 449 proteins were upregulated and 443 proteins were downregulated, compared with pruned tea plants. A DEP volcano was used to show differentially expressed proteins in leaves of pruned versus unpruned plants (Figure S1), FC of each protein (log2 pruned/unpruned) was represented on the horizontal axis, and *p* (-log10 value) was represented on the vertical axis. A hierarchical cluster was conducted to analyze the expression levels in the two groups (Figure 2A). GO term enrichment analysis showed that “photosynthesis” and “transmembrane transport” were the higher proportion and significantly enriched differentially expressed proteins under the “biological process” category; moreover, “transporter activity” and “transmembrane transporter activity” were the higher proportion and significantly enriched proteins under the “molecular function” category. Additionally, “integral component of membrane” and “intrinsic component of membrane” were the higher proportion and significantly enriched differentially expressed proteins under the “cellular component” category (Figure 2B). Moreover, results of the KEGG pathway-enrichment analysis showed that “ribosome” and “photosynthesis” were the most significantly enriched pathways (Figure S2), indicating that DEPs in those pathways may contribute significantly more to the formation of different quality components present in the leaves of pruned and unpruned tea plants.

### 3.3. Catechin Content Decreased in Fresh Leaves of Long-Term Unpruned Tea Plants, and Most Enzymes Involved in Catechins Biosynthesis Were Downregulated

**Figure 3:**
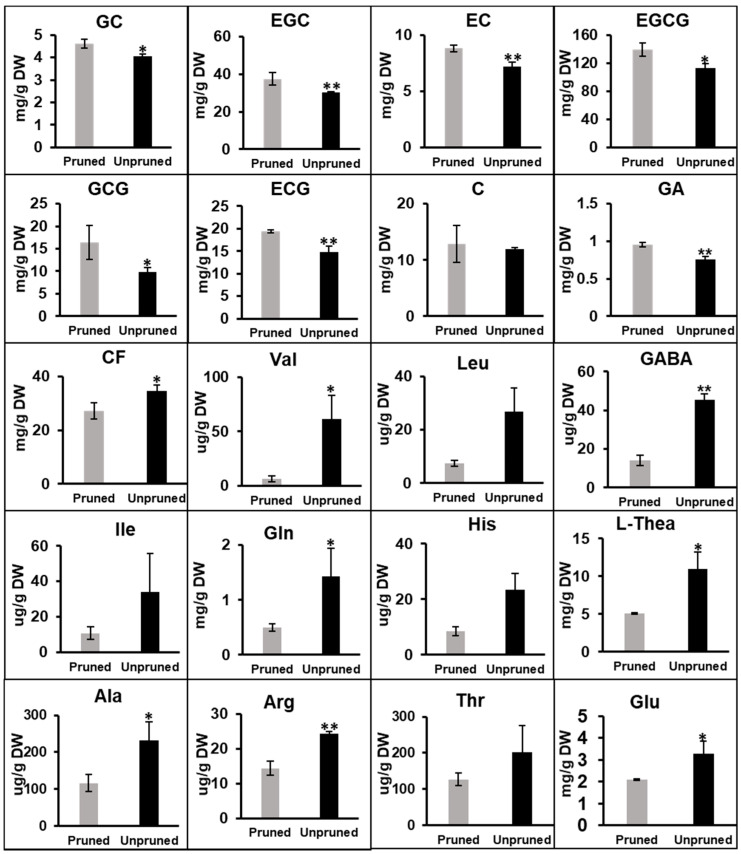
Concentration of catechins, gallic acid, caffeine and amino acids in leaves of pruned and unpruned tea plants. Data is presented as mean ± SD. * represents significant difference (*p* < 0.05), ** represents extremely significant difference (*p* < 0.01). C, catechin; CF, caffeine; EC, epicatechin; ECG, epicatechin gallate; EGC, epigallocatechin; EGCG, epigallocatechin gallate; GA, gallic acid; GC, gallocatechin; GCG, gallocatechin gallate; Ala, alanine; Arg, arginine; GABA, gamma-aminobutyric acid; Gln, glutamine; Glu, Glutamate; His, histidine; Ile, isoleucine; Leu, leucine; L-Thea, theanine; Thr, Threonine; Val, valine.

**Figure 4:**
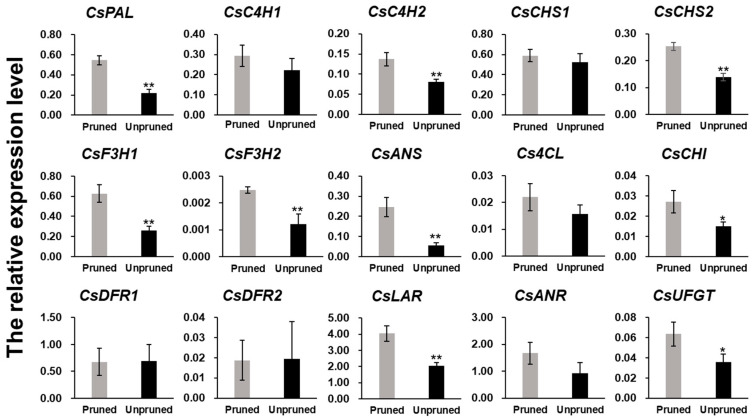
Relative expression levels of candidate genes by quantitative real-time PCR. The data were analyzed by *t* test, * represents significant difference (*p* < 0.05), ** represents extremely significant difference (*p* < 0.01). 4CL, 4-coumaroyl-CoA ligase; ANR, anthocyanidin reductase; ANS, anthocyanidin synthase; C4H, cinnamate; CHI, chalcone isomerase; CHS, chalcone synthase; DFR, dihydroflavonol 4-reductase; F3H, flavonoid 3-hydroxylase; LAR, leucoanthocyanidin reductase; PAL, phenylalanine ammonia; UFGT, UDP- glucose flavonoid 3-o-glucosyl transferase.

Catechins are abundant in buds and leaves of tea plants and contribute to the bitterness and astringency of tea infusions. Tea catechins comprise catechin (C), gallocatechin (GC), epicatechin (EC), epigallocatechin (EGC), as well as their gallate esters: gallocatechin-3-gallate (GCG), epicatechin-3-gallate (ECG), and EGCG [31]. In this study, catechin content in fresh tea leaves was determined. Except for C, content of other types of catechins, such as EGC, EC, and ECG, was significantly lower in leaves of unpruned, compared to content of those of pruned tea plants; moreover, GC, EGCG, and GCG contents were much lower in the leaves of long-term unpruned tea plants (Figure 3). Content of gallic acid (GA), the precursor of ester catechin synthesis, was also significantly higher in leaves of pruned tea plants. Catechins are biosynthesized through the flavonoid and phenylpropanoid pathways; further, phenylalanine ammonia lyase (PAL) is the first key enzyme responsible for catalyzing L-phenylalanine deamination to trans-cinnamic acid [19]. Our proteomic analysis identified 35 proteins involved in catechin biosynthesis, eight of which were downregulated (fold change < 0.85, *p* < 0.05) in leaves of long-term unpruned tea plants, while none of the other 27 proteins were upregulated significantly (Table 1 and Table S2). Among the eight downregulated proteins, three phenylalanine ammonia-lyases (A0A286QXV0, A0A286QXV1, and P45726) were identified. It has been reported that the expression of PAL corresponds with catechin content in tea plants [32]. Cinnamate-4-hydroxylase, chalcone synthase, flavanone 3-hydroxylase (F3H), and anthocyanidin synthase (ANS), all of which are involved in the catechin biosynthesis pathway, were downregulated in leaves of long-term unpruned tea trees. The downregulated expression of these proteins may decrease the synthesis of catechins in unpruned tea leaves. Catechin biosynthesis-related gene expression was determined via qRT-PCR. As shown in Figure 4, the expression levels of most genes decreased in tea leaves of unpruned trees, compared to those in leaves of pruned trees. The expression levels of CsPAL, CsF3H1, CsF3H2, and CsANS were 2.49-, 2.40-, 2.04-, and 4.43-fold lower in unpruned tea trees, respectively. These results confirmed the accuracy of proteomic data and verified that these genes were related to the fungicidal activity of catechins. Based on the proteomics and gene expression analysis, the results indicated that relatively lower expression of key genes and proteins in catechin metabolism pathway is the main reason for the decreased catechin content in unpruned tea plants.

### 3.4. Most Free Amino Acids Were More Abundant in Fresh Leaves of Long-Term Unpruned Tea Plants

Amino acids comprise another kind of metabolite closely related to tea quality. It has been reported that amino acids contribute to tea freshness and mellowness, and high amino acid content is reportedly the most important characteristic of high-quality green tea [33,34]. In this study, free amino acids in fresh tea leaves were determined using UPLC-MS, and 22 free amino acids were detected in leaves of both pruned and unpruned tea trees. Overall, the free amino acid content was generally higher in the leaves of unpruned tea plants. Twelve amino acids were over 1.5-fold higher in tea leaves of unpruned trees than in those of pruned trees (Figure 3; Table S3). Content of theanine, the most abundant free amino acid, can reach 10,970.12 ug/g in leaves of unpruned trees, while reaching only 5100.46 ug/g in those of pruned tea trees. L-Theanine contributes to the freshness and umami flavor in tea infusions, and generates a pronounced caramel aroma [35,36]. The biosynthesis of L-theanine from L-glutamate and ethylamine is catalyzed by L-theanine synthetase (TS, EC 6.3.1.6), which is highly homologous to L-glutamine synthetase (GS) [37,38]. In our proteomic results, six GS proteins were identified in leaves of both pruned and unpruned tea plants, and two of them were annotated as L-theanine synthase (TS). However, there was no significant difference in the protein expression of the six GSs (Table S2). Previous studies reported that L-theanine in tea plants was mostly synthesized in the roots and then transported to buds and growing leaves [39]. Therefore, it was speculated that the significant difference in leaf theanine content between pruned and unpruned tea trees might be caused by the difference in theanine synthesis occurring in the roots. The expression levels of proteins involved in glutamine and GABA synthesis in leaves did not differ significantly between pruned and unpruned tea plants. In tea, L-theanine and glutamine are conducive to the formation of volatile compounds and contribute to aromatic quality. The relatively higher amino acid content in leaves of unpruned tea trees may be an important reason for its excellent aroma and taste.

### 3.5. Characteristic Floral and Honey Aroma Compounds Accumulated in the Leaves of Unpruned Tea Plants

In this study, we compared the difference in volatiles profiles of tea leaves between unpruned and pruned tea plants. Twenty representative aroma components were analyzed by GC-MS and quantified by reference to standard compounds. Four aroma components in fresh tea leaves and seven aroma components in tea products were significantly higher in unpruned tea trees (Table 2, Figure S3). The characteristic aroma of the tea products is an important criterion in the evaluation of tea quality. Unique floral and honey flavor are the main characteristics of Chaozhou Dancong tea, and trans-beta-ocimene, methyl salicylate, benzeneacetaldehyde, trans-linalool oxide (furanoid), benzyl alcohol, jasmine lactone, and trans-nerolidol were the representative floral and honey aroma compounds found [40,41]. Metabolomics results showed that the contents of these seven aroma components in tea products from unpruned tea plants were significantly higher than those in pruned tea plants (Table 2). This may be the main reason for unpruned tea leaves having higher sensory evaluation scores for aroma than pruned tea leaves. Four aromatic compounds, methyl salicylate, 3-Hexen-1-ol, alpha-Farnesene, and cis-Geraniol, were significantly higher in content, in fresh tea leaves from unpruned tea trees. However, no DEPs related to the synthesis of these aroma components in tea were detected through proteomic analysis.

### 3.6. Proteins Involved in Chlorophyll Biosynthesis and Light-Dependent Reactions of Photosynthesis Accumulated in Unpruned Tea Plants

Chlorophylls play essential roles in photosynthesis, light harvesting, and light energy transduction. The levels of chlorophyll a and chlorophyll b are important indexes used to evaluate photosynthetic capacity [47]. In this study, the concentrations of chlorophyll a and chlorophyll b in fresh leaves of pruned and unpruned tea trees were determined. Compared with pruned tea trees, the chlorophyll concentrations in leaves of unpruned tea trees were significantly higher (Figure 1E). Wei et al. (2011) reported that chlorophyll a formation in tea leaves was positively correlated with epicatechin and epigallocatechin content during young leaf development [15]. However, there was no significant correlation between chlorophyll and catechin contents in our results. During early spring, development of chloroplasts from etioplasts and the accumulation of chlorophyll a and chlorophyll b occurs in the new shoots of tea plants. Chlorophyll biosynthesis is a very complex process, occurring via a series of coordinated reactions, catalyzed by numerous enzymes [48]. As shown in Table 1, five enzymes involved in chlorophyll biosynthesis were positively identified, including three NADPH-protochlorophyllide oxidoreductases (PORs), a cytochrome c oxidase assembly protein subunit 15, and a heme oxygenase. POR is the key enzyme that catalyzes protochlorophyllide to chlorophyllide, which is ultimately converted to chlorophyll in developing leaves. All these five enzymes were upregulated in the leaves of unpruned tea plants, and this might be the main reason for the relatively higher chlorophyll content in this case.

Eleven photosynthesis-related DEPs, identified as involved in light-dependent reactions of photosynthesis, were upregulated in leaves of unpruned tea plants (Table 1). Four nucleus-encoded chloroplast proteins, known as oxygen-evolving enhancer (OEE) proteins, were identified, and it has been reported that OEE proteins play an important role in the light-induced oxidation of water in photosystem II (PSII) in plants [49]. Furthermore, we identified two other subunits of PSII, namely, PsbP and Psb27, which are reportedly essential for plant photoautotrophic growth and the recovery process of PSII, respectively [50,51]. Plastocyanin and 2Fe-2S ferredoxin-type domain-containing protein are two photosynthetic electron transfer proteins. The upregulation of these two proteins may modulate the rapid transfer of electrons from PSII to photosystem I, thereby affecting the formation of the transmembrane electrochemical proton gradient for generating ATP and NADPH. Three identified ATP synthase subunits were upregulated in the fresh leaves of unpruned tea plant, and those subunits were involved in hydrogen ion transmembrane-transporter activity. Energy supply is important for the synthesis of various metabolites, and upregulation of proteins involved in photosynthesis may ultimately enhance energy transformation during photosynthesis. Therefore, we speculate that the upregulated expression of photosynthesis-related proteins might provide more energy for the synthesis of quality components in the leaves of unpruned tea trees.

## 4. Conclusions

**Figure 5:**
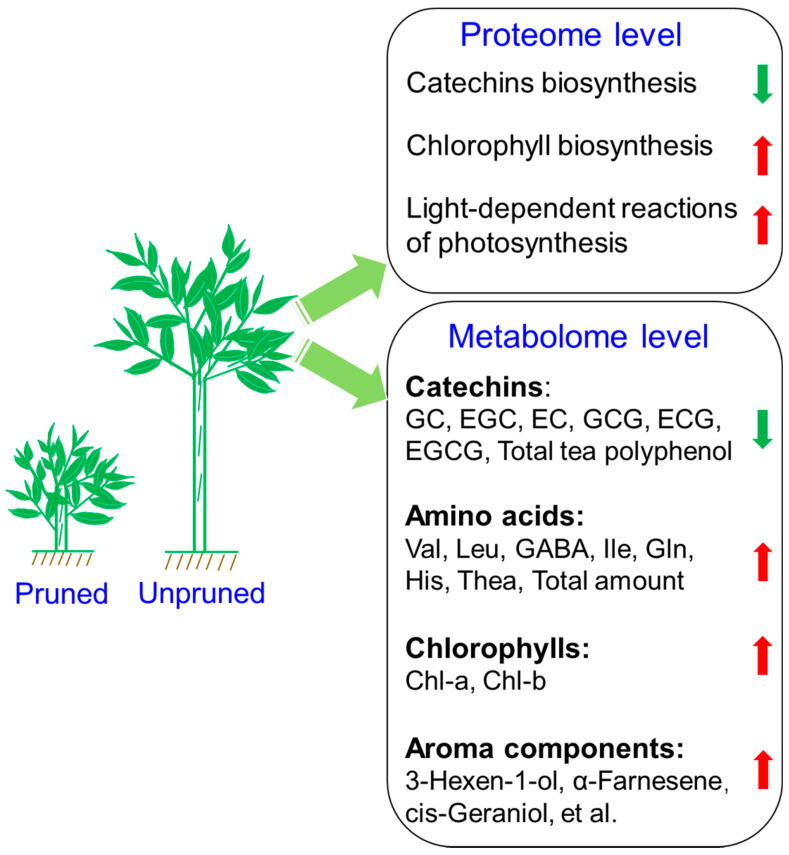
Summary on the formation of different quality constitutes in leaves of pruned and unpruned tea plants. Red arrow represents the increasing tendency in leaves from long-term unpruned ‘Dancong’ tea plant, while green represents the decreasing tendency.

In this study, taste and aroma of tea products from long-term unpruned and pruned tea plants were compared via sensory evaluation. The results showed that aroma and taste of tea products from unpruned plants were superior to those of the corresponding counterparts from pruned plants. Metabolomics results showed that catechin contents were relatively lower in tea leaves from unpruned plants, while free amino acids, chlorophylls, and characteristic floral and honey aroma compounds accumulated in the leaves of unpruned tea plants. TMT based proteomics was used to analyze the mechanism of formation of differential quality components in their fresh leaves. In long-term unpruned ‘Dancong’ tea plants, the downregulated expression of enzymes in the catechin synthesis pathway was an important reason for the relatively lower catechin content. Proteins involved in chlorophyll biosynthesis and light-dependent reactions of photosynthesis accumulated in leaves of unpruned tea plants, and the consequent enhancement of photosynthesis was beneficial to the accumulation of free amino acids and aroma components in tea leaves (Figure 5). Therefore, our study provides novel observations, with respect to the mechanism of formation of different tea-quality constituents in unpruned and long-term pruned tea plants.

## Figures and Tables

**Table 1:** Differentially accumulated proteins related to biosynthesis of quality constitutes in tea leaves between long-term unpruned and pruned tea plants.

Acc. No. ^a^	Protein Name	Gene Name ^b^	%cov ^c^	MW/Pi ^d^	Nup ^e^	Ratio ^f^ (Means ± SD)	*p* ^g^
**Catechin metabolism**							
A0A286QXV0	Phenylalanine ammonia-lyase	*PALc*	34.88	77.32/6.65	7	−1.28 ± 0.07	0.02
A0A286QXV1	Phenylalanine ammonia-lyase	*PALb*	28.17	76.59/6.14	3	−1.28 ± 0.05	0.00
P45726	Phenylalanine ammonia-lyase	*PAL*	30.81	77.70/6.58	9	−1.22 ± 0.06	0.02
A0A286QXW9	Cinnamate-4-hydroxylase	*C4Ha*	31.88	58.12/9.26	5	−1.20 ± 0.07	0.02
M4VNY3	Chalcone synthase	*CHS3*	29.82	42.76/6.55	5	−1.20 ± 0.06	0.01
A0A286QXX8	Flavanone 3-hydroxylase	*F3Hb*	21.35	39.95/5.78	3	−1.39 ± 0.10	0.02
A0A0S2E967	Anthocyanidin synthase (Fragment)	*ANS*	15.91	34.80/5.73	1	−1.20 ± 0.05	0.02
A0A5B9G8C2	Caffeoyl shikimate esterase	*CSE*	6.46	36.55/5.54	2	−1.22 ± 0.06	0.01
**Chlorophyll metabolism**							
A0A4S4ED58	NADPH-protochlorophyllide oxidoreductase	*por*	30.33	43.53/8.88	7	1.23 ± 0.03	0.00
A0A1L5JJ09	NADPH-protochlorophyllide oxidoreductase	*por*	47.29	44.18/8.59	1	1.26 ± 0.14	0.02
A0A4S4DWI4	NADPH-protochlorophyllide oxidoreductase	*por*	33.63	49.46/8.51	11	1.36 ± 0.06	0.01
A0A4S4DM53	cytochrome c oxidase assembly protein subunit 15	*COX15*	2.96	52.29/10.07	1	1.37 ± 0.04	0.05
A0A4S4EC13	heme oxygenase (biliverdin-producing, ferredoxin) [EC:1.14.15.20]	*HO*	18.79	32.42/8.56	5	1.49 ± 0.31	0.00
**Photosynthesis**							
G3F4I6	Oxygen-evolving enhancer protein	*psbO*	51.95	35.26/7.99	8	1.21 ± 0.03	0.01
A0A4S4ES74	photosystem II oxygen-evolving enhancer protein 1	*psbO*	37.39	46.37/4.91	9	1.30 ± 0.01	0.00
A0A4V3WK35	photosystem II oxygen-evolving enhancer protein 3	*psbQ*	42.49	24.71/9.72	11	1.30 ± 0.03	0.03
A0A4S4DBK0	photosystem II oxygen-evolving enhancer protein 3	*psbQ*	7.24	24.81/9.44	2	1.23 ± 0.10	0.00
A0A4V3WQ43	PsbP domain-containing protein	*psbP*	48.69	28.53/8.10	11	1.27 ± 0.03	0.00
A0A4S4DRK6	photosystem II Psb27 protein	*psb27*	26.86	19.05/9.83	5	1.41 ± 0.07	0.00
A0A4S4EUK1	Plastocyanin	*petE*	38.24	17.15/5.20	3	1.23 ± 0.07	0.00
A0A4S4EQ68	2Fe-2S ferredoxin-type domain-containing protein	*petF*	31.62	14.87/7.20	3	1.24 ± 0.08	0.01
A0A4S4DQD2	F-type H^+^-transporting ATPase subunit b	*ATPF0B*	40.37	24.00/6.89	10	1.44 ± 0.26	0.00
L0E5W5	ATP synthase subunit b, chloroplastic	*ATPF0B*	42.39	20.99/9.04	10	1.25 ± 0.13	0.00
A0A4S4F0J0	F-type H+-transporting ATPase subunit delta	*ATPF1D*	35.2	26.88/9.19	9	1.22 ± 0.13	0.00

^a^ Accession numbers in Uniprot database. ^b^ Gene name in Uniprot database. ^c^ Percent sequence coverage of identified proteins. ^d^ Protein MW/pI, MW (kDa) of predicted protein/pI of predicted protein. ^e^ Nup, number of unique peptides identified for each protein. *^f^* Ratio, the ratio between intensities of identified protein species in tea leaves of unpruned to pruned tea plants. The data represents mean ± SD (standard deviation) of three independent biological replicates. Significant changes are indicated by boldface. ^g^ *p*-value, which indicates the significance of the increased and decreased expression of proteins according to a Student’s *t*-test.

**Table 2:** Concentrations of volatile compounds in fresh tea leaves and tea products from long-term unpruned and pruned ‘Dancong’ tea plants.

Compound Name	Rt (min)	Fresh Tea Leaves (nmol/g DW)		Tea Products (nmol/g DW)		Odour Description	Reference
		Pruned	Unpruned	Pruned	Unpruned		
trans-beta-Ocimene	9.29	2.62 ± 0.96	2.61 ± 1.57	0.87 ± 0.32	9.76 ± 1.24	Floral, roasted	[41]
Methyl salicylate	26.22	57.44 ± 6.18	98.75 ± 18.83	1.60 ± 0.25	7.56 ± 1.17	Sweet	[42]
Benzeneacetaldehyde	22.14	12.07 ± 1.58	6.10 ± 1.89	1.98 ± 0.70	5.83 ± 4.28	Honey-like	[42]
trans-Linalool oxide (furanoid)	15.8	38.31 ± 9.65	40.16 ± 8.42	5.13 ± 0.32	11.83 ± 5.18	Sweet, floral, citrus	[43,44]
Benzyl alcohol	28.845	7.82 ± 0.84	7.76 ± 0.54	3.77 ± 1.74	7.13 ± 1.92	Rose	[40]
Jasmine lactone	38.88	4.21 ± 0.24	4.27 ± 0.18	11.70 ± 1.01	19.35 ± 4.45	Floral	[40]
trans-Nerolidol	33.455	10.33 ± 1.47	10.34 ± 1.33	11.25 ± 1.26	18.37 ± 1.87	Floral (rose), apple, green	[40]
3-Hexen-1-ol, (Z)-	13.76	27.21 ± 8.99	146.87 ± 4.42	0.25 ± 0.03	0.27 ± 0.02	Grassy	[42]
alpha-Farnesene	24.75	17.38 ± 3.85	34.83 ± 4.67	93.56 ± 6.01	98.49 ± 12.78	Sweet, mild	[45]
cis-Geraniol	26.84	173.95 ± 14.98	343.48 ± 87.00	11.60 ± 0.64	14.03 ± 4.32	Rose-like	[40]
3-Hexen-1-ol, acetate, (Z)-	11.77	143.82 ± 20.78	228.64 ± 7.36	-	-	fresh-green odors	
Acetic acid, 2-phenylethyl ester	28.775	0.33 ± 0.02	0.33 ± 0.01	0.12 ± 0.01	0.14 ± 0.04		
Benzyl nitrile	30.365	3.50 ± 0.47	2.67 ± 0.60	1.61 ± 0.21	1.72 ± 0.70		
3,5-Dimethoxytoluene	29.745	0.35 ± 0.02	0.35 ± 0.01	0.13 ± 0.01	0.13 ± 0.01		
Nerolidol	32.4	1.79 ± 0.10	1.76 ± 0.06	0.70 ± 0.10	0.73 ± 0.14	Floral (rose), apple, green	[40]
Linalool	19.195	202.15 ± 24.52	197.03 ± 24.56	2.85 ± 0.67	1.92 ± 0.62		
2-Hexanol	8.61	0.00 ± 0.03	0.00 ± 0.02	0.00 ± 0.04	0.00 ± 0.17	Fresh-green odors	[46]
1-Hexanol	12.76	0.00 ± 0.10	1.23 ± 0.51	0.00 ± 0.06	-	Fresh-green odors	[46]
Benzaldehyde	18.405	1.61 ± 1.01	0.26 ± 0.34	-	-	Almond	[42]

## Data Availability

All data is contained within the article.

## Supplementary Materials

The following are available online at https://www.mdpi.com/article/10.3390/foods10112649/s1, Table S1: The primers used for quantitative real time PCR (qRT-PCR) in the study; Table S2: Differentially accumulated proteins related to biosynthesis of quality constitutes in tea leaves between long-term unpruned and pruned tea plants; Table S3: Differentially accumulated free amino acids in tea leaves between long-term unpruned and pruned tea plants; Figure S1: A DEP volcano for differentially expressed proteins in leaves of pruned versus unpruned tea plants; Figure S2: KEGG pathways (TOP 20) for differentially expressed proteins in tea leaves of pruned and unpruned plants. Figure S3: GC-MS chromatograms of aroma compound in tea leaves of unpruned and pruned ‘Dancong’ tea plants.

## Abbreviations

ANS	Anthocyanidin synthase
C	Catechin
DEP	Differentially expressed protein
DW	Dry weight
EC	(Epicatechin
ECG	Epicatechin-3-gallate
EGC	Epigallocatechin
EGCG	Epigallocatechin gallate
F3H	Flavanone 3-hydroxylase
GA	Gallic acid;
GC	Gallocatechin;
GCG	Gallocatechin-3-gallate;
GC-MS	Gas chromatography–mass spectrometry;
GO	Gene Ontology;
GS	L-glutamine synthetase
KEGG	Kyoto Encyclopedia of Genes and Genomes
MS	Mass spectrometry
MS/MS	Tandem mass spectrometry;
PAL	Phenylalanine ammonia lyase;
POR	Protochlorophyllide oxidoreductase;
PSII	Photosystem II
qRT-PCR	Quantitative real time polymerase chain reaction
TMT	Tandem mass tag
UPLC	Ultra-performance liquid chromatography
