# Supplementary material for: Effects of Long-Term Non-Pruning on Main Quality Constituents in ‘Dancong’ Tea (Camellia sinensis) Leaves Based on Proteomics and Metabolomics Analysis
Source: Foods. 2021 Nov 1;10(11):2649. doi: 10.3390/foods10112649 (PMC8625003; doi:10.3390/foods10112649)
Supplement: Supplementary file 1 [file foods-10-02649-s001.zip › foods-1396477-supplementary.pdf]

Supplementary Materials

Table S1. The primers used for quantitative real time PCR (qRT-PCR) in the study.

Gene	Accession number	Forward primer 5'-3'	Reverse primer 5'-3'
CsPAL	KY615669.1	CCGTTCAAGCAAGCAGT	ACATTGTAGCCCTCGTAGA
CsC4H1	KY615675.1	CGATAGAATGGGGCATAGCA	TGGAGGTAGGGGAGTTTGTAGG
CsC4H2	KY615676.1	GCTCGGCAGCTATGACATCC	CTCCTCCTACCAACACCGAATG
CsCHS1	KY615681.1	TGAAGGACCTGCCACGGTTATG	GCCTTATGCTCGCTGTTTGT
CsCHS2	KY615683.1	GGCAACCCCACCAAACT	CCACCTTATGCTCGCTATTA
CsF3H1	KY615688.1	ACAACAACGCTTACGGCTCTC	AAACCCCCCAACCTTCACA
CsF3H2	KY615689.1	TCAAACGCCACACAGACCC	CAACGAAAGCCCCCTCAA
CsANS	AY830416.1	GGCCACAAGTGCCTACAATTG	CCCATGATTCACCAAATGCA
Cs4CL	DQ194356.1	GGAGGTTATCCTGGACCTCA	GGCAAGCCTTGTAGTGTGAA
CsCHI	DQ904329.1	AGATTTCTCGGCTTCCAG	CTCTTTATGCTTCCTTGTCA
CsDFR1	KY615690.1	ATTGGCAGAGAAAGCAGCAT	GTGATTAGGCTTGGTGGGAA
CsDFR2	KY615691.1	AATGGTTATGGTGGTGGCG	CGAAATTCATGGGAGTGGC
CsLAR	KY615698.1	AAAAGAGGAGGGTGCGG	GGAACTCATCCAAAGGGGG
CsANR	KY615701.1	GAGTACTTCAAGGCTAAGGGG	CAAGCAAACCAAGCAAAACC
CsUFGT	KP682359.1	GGCAAGAAGCTAATAGGGTCGTT	TTGTATCATTCGGAAGTGgTGGG
CsEF-1α	XM_028247179.1	TTGGACAAGCTCAAGGCTGAACG	ATGGCCAGGAGCATCAATGACAGT

4CL, 4-coumaroyl-CoA ligase; ANR, anthocyanidin reductase; ANS, anthocyanidin synthase; C4H, cinnamate; CHI, chalcone isomerase; CHS, chalcone synthase; DFR, dihydroflavonol 4-reductase; EF1, encoding elongation factor 1α; F3H, flavonoid 3-hydroxylase; LAR, leucoanthocyanidin reductase; PAL, phenylalanine ammonia; UFGT, UDP- glucose flavonoid 3-o-glucosyl transferase.

Table S2. Differentially accumulated proteins related to biosynthesis of quality constitutes in tea leaves between long-term unpruned and pruned tea plants.

See the attached Excel file.

Table S3. Differentially accumulated free amino acids in tea leaves between long-term unpruned and pruned tea plants.

Amino acid μg/g (Dry weight)	Pruned	Unpruned
Val	6.30 ± 2.85	61.26 ± 22.34
Leu	7.48 ± 1.14	26.87 ± 18.82
GABA	14.19 ± 2.63	45.29 ± 3.08
Ile	10.82 ± 3.72	34.11 ± 21.73
Gln	497.93 ± 67.94	1421.49 ± 521.47
His	8.51 ± 1.60	23.41 ± 11.91
L-Thea	5100.46 ± 98.53	10970.12 ± 2283.67
Ala	116.22 ± 22.64	231.50 ± 49.62
Arg	14.45 ± 1.98	24.39 ± 0.51
Thr	126.72 ± 17.52	202.90 ± 72.39
Glu	2102.77 ± 28.81	3279.37 ± 570.75
Pro	16.29 ± 1.60	24.79 ± 3.62
Tyr	17.65 ± 1.53	25.82 ± 6.58
Asp	328.37 ± 52.58	442.62 ± 102.73
Lys	32.50 ± 3.13	43.77 ± 9.88
Ser	282.88 ± 14.17	372.69 ± 117.05
Phe	28.55 ± 1.39	36.75 ± 8.21
Orn	0.90 ± 0.17	1.15 ± 0.57
Gly	33.41 ± 8.57	40.80 ± 14.17
Met	4.75 ± 0.76	5.55 ± 0.32
Asn	18.90 ± 2.24	20.38 ± 2.11
Trp	54.67 ± 13.34	49.09 ± 2.43

Data are expressed as mean ± S.D. (n=3). Val, valine; Leu, leucine; GABA, gamma-aminobutyric acid; Ile, isoleucine; Gln, glutamine; His, histidine; L-Thea, theanine; Ala, alanine; Arg, arginine; Thr, Threonine; Glu, Glutamate; Pro, proline; Tyr, Tyrosine; Asp, Aspartic acid; Lys, lysine; Ser, Serine; Phe, Phenylalanine; Orn, Ornithine; Gly, Glycine; Met, Methionine; Asn, Asparagine; Trp, Tryptophan.

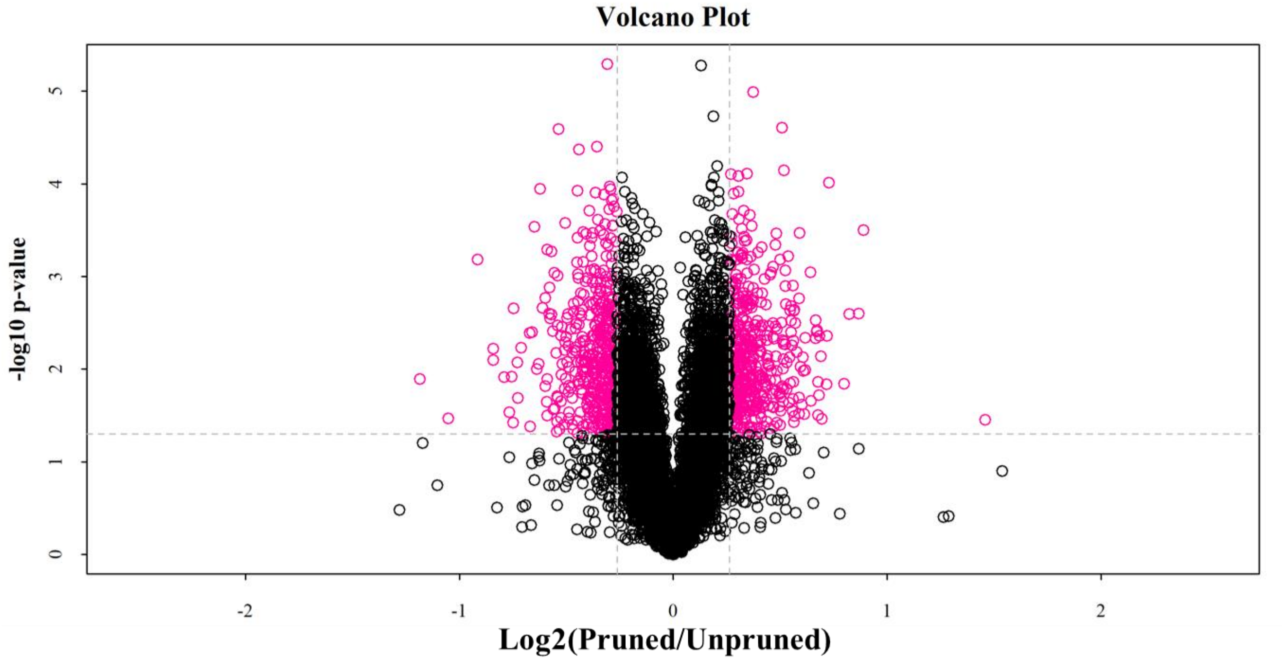

Figure S1. A DEP volcano for differentially expressed proteins in leaves of pruned versus unpruned tea plants.

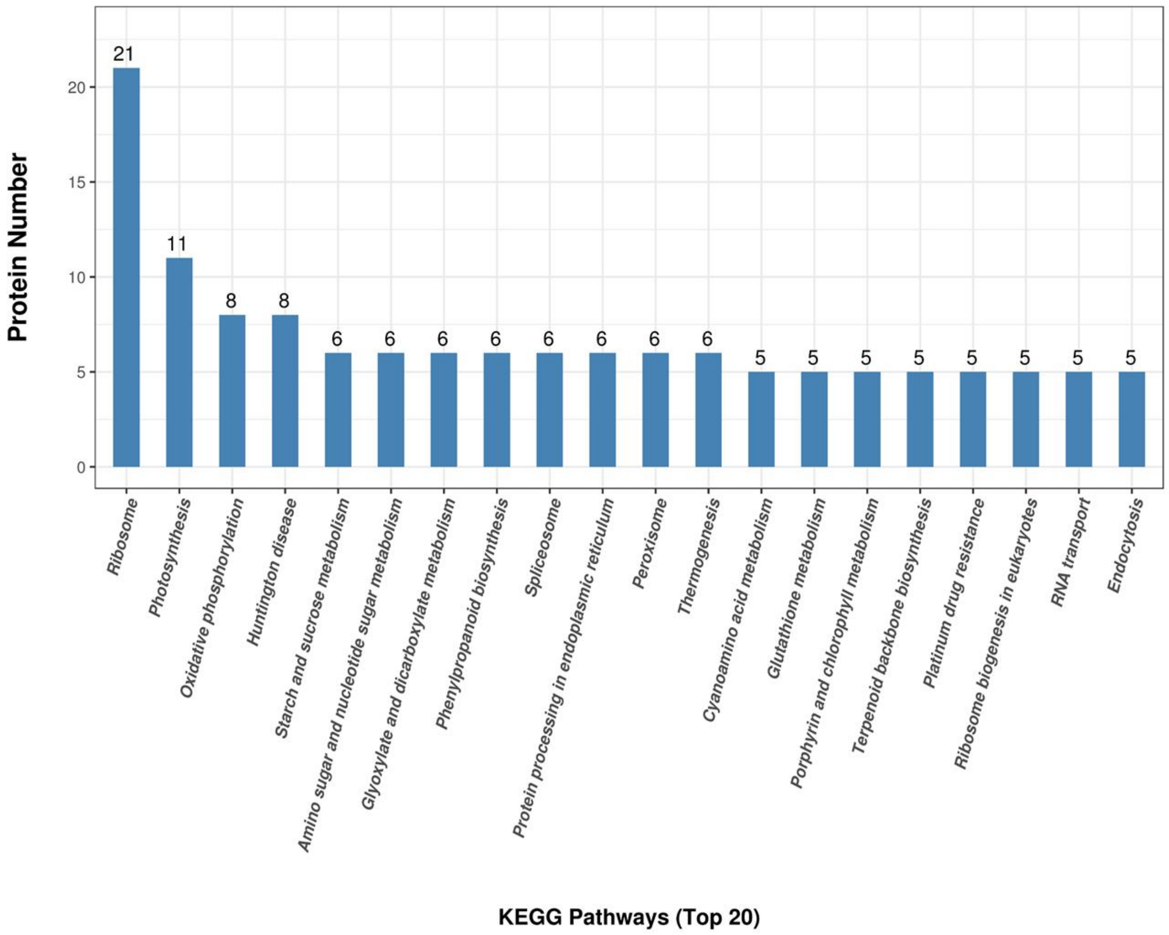

Figure S2. KEGG pathways (TOP 20) for differentially expressed proteins in tea leaves of pruned and unpruned plants.

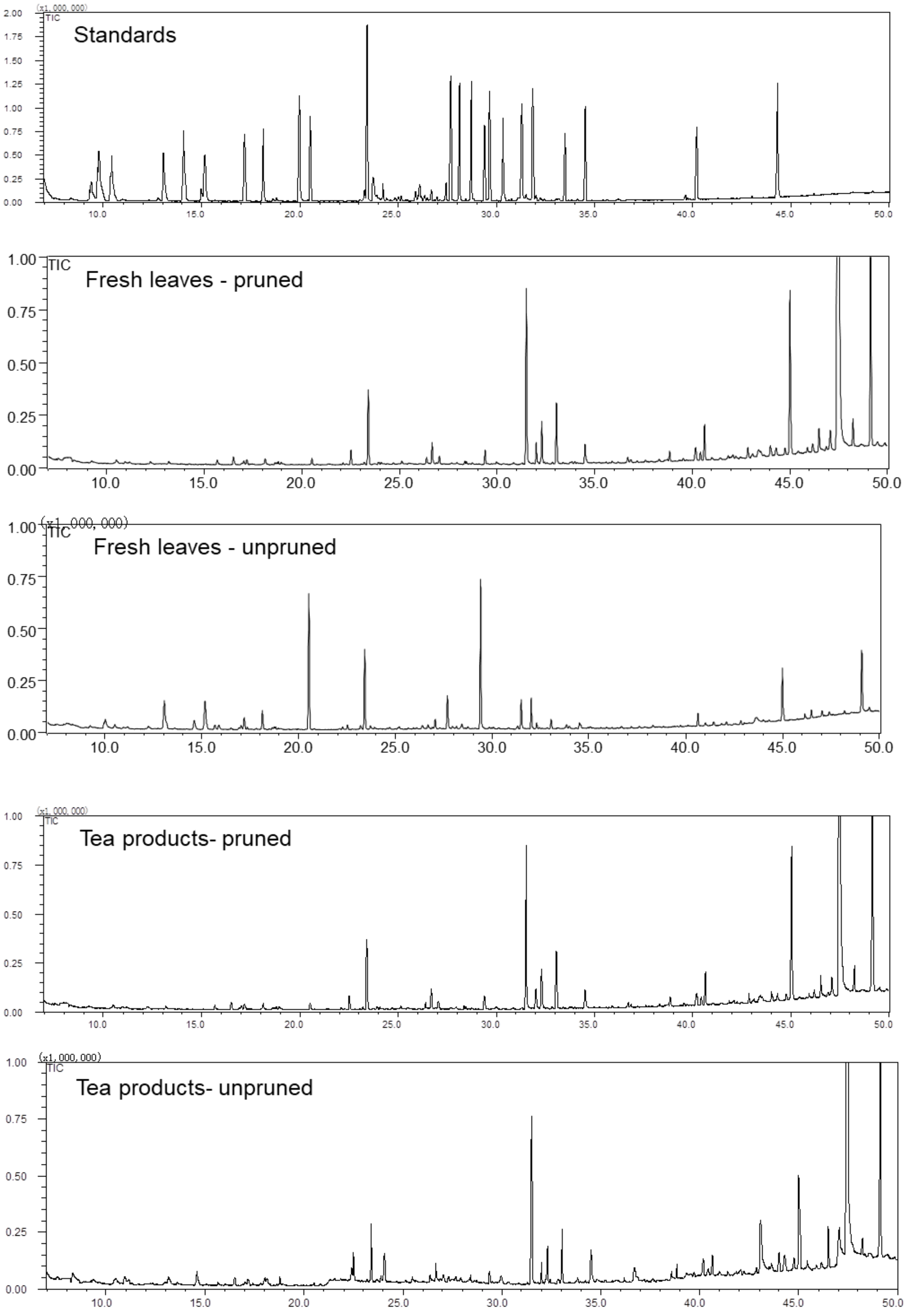

Figure S3. GC-MS chromatograms of aroma compound in tea leaves of unpruned and pruned 'Dancong' tea plants
